# Preparation of Deer Brain Peptide Chelated with Zinc and Its Effect on Improving Memory Impairment in Insomnia Mice Induced by Para-Chlorophenylalanine

**DOI:** 10.3390/nu18152462

**Published:** 2026-07-28

**Authors:** Jiapeng Song, Ran Ning, Yike Du, Junkoo Yi, Zhongmei He, Jia Zhou, Xuezhen Li, Weijia Chen

**Affiliations:** 1 College of Chinese Medicinal Materials, Jilin Agricultural University, Changchun 130118, China; 15637212726@163.com (J.S.); 18943978448@163.com (R.N.); 17837201061@163.com (Y.D.); heather78@126.com (Z.H.); 007152@jlau.edu.cn (J.Z.);; 2 Division of Animal Science, School of Animal Life Convergence Sciences, Anseong City 12646, Republic of Korea; junkoo@hknu.ac.kr; 3 Jilin Provincial Engineering Research Center for Efficient Breeding and Product Development of Sika Deer, Changchun 130118, China

**Keywords:** peptide, insomnia, chelation, memory impairment

## Abstract

**Background/Objectives**: Chronic insomnia commonly induces progressive memory decline, severely compromising human daily life and work capability. At present, there are no safe long-term available agents that can concurrently relieve insomnia symptoms and rescue accompanying memory dysfunction. This study aimed to optimize the preparation of deer brain peptides (DBPP) and zinc-chelated DBPP (Zn-DBPP), and explore their protective effects and molecular mechanism against insomnia-caused memory impairment, hoping to develop novel functional candidates for related neurological disorders. **Methods**: Single-factor experiments combined with response surface methodology were used to optimize the synthesis process of DBPP and Zn-DBPP. A para-chlorophenylalanine-induced insomnia mouse model was established. The structural characteristics, amino acid composition, and antioxidant activity of the products were verified via multiple spectroscopic and biochemical assays. Pentobarbital sodium sleep test and Morris water maze test assessed behavioral changes. Hippocampal neuronal morphology and BDNF-TrkB pathway expression were detected by histological staining, immunofluorescence and Western blotting. **Results**: The optimized DBPP achieved a hydrolysis rate of 43.89%, and Zn-DBPP possessed a zinc content of 143.37 mg/g. Successful zinc chelation, rich amino acid components, and strong antioxidant capacity were confirmed in Zn-DBPP. In vivo results showed that Zn-DBPP elevated brain zinc levels, improved learning and memory deficits, and restored hippocampal neuronal damage in insomniac mice. Mechanically, Zn-DBPP alleviated memory impairment by upregulating the BDNF-TrkB signaling pathway. **Conclusions**: The optimized Zn-DBPP exhibits excellent neuroprotective effects against insomnia-induced memory dysfunction. This work provides a reliable theoretical basis for the application of Zn-DBPP as a promising functional food or drug candidate for intervening in insomnia and cognitive decline.

## 1. Introduction

As social pressures continue to escalate in the modern world, insomnia has emerged as a significant public health concern. Globally, approximately 16.2% of the population suffers from insomnia, with severe cases accounting for 7.9% [1]. China, the United States, and Brazil rank among the countries with the highest prevalence of insomnia [2]. Insomnia not only compromises next-day mood and cognitive performance but also contributes to long-term memory impairment [3]. The core mechanism underlying insomnia-induced memory impairment involves sleep deprivation, which impairs hippocampal synaptic structural plasticity—manifested as reduced dendritic spine density and synaptic efficacy—and disrupts functional connectivity among memory-related regions (e.g., hippocampus, amygdala, and prefrontal cortex). Consequently, this interferes with the normal processing of various memory types [4,5,6]. Currently, the commonly used medications for insomnia on the market include estazolam and flurazepam, while the drug frequently used to treat memory impairment is oxiracetam, among others [7]. However, these drugs are specifically designed to treat insomnia or memory disorders, and long-term use may lead to certain dependence and toxic side effects [8]. Nevertheless, in clinical practice, insomnia and memory disorders frequently co-occur, often necessitating the use of at least two separate medications to manage each condition. This highlights the urgent need for a single therapeutic agent or health supplement that possesses low toxicity and minimal side effects, while effectively treating insomnia and ameliorating memory impairment.

Deer brain is highly valued in traditional Chinese medicine (TCM) for its therapeutic properties. Historically, it has been employed to alleviate insomnia and cognitive impairment. In the Ming Dynasty pharmacopeia Compendium of Materia Medica (Bencao Gangmu), deer brain is recorded as being able to “dispel mental restlessness and tranquilize the five viscera.” The term “mental restlessness” in traditional medicine refers to symptoms such as insomnia, frequent nightmares, and mental fatigue. Given its established use in TCM, deer brain presents considerable potential as a raw material for developing novel therapeutics aimed at ameliorating memory deficits induced by chronic insomnia.

Peptides are small-molecule active substances produced by the enzymatic hydrolysis of proteins. It offers advantages such as low toxicity, minimal side effects, and good absorbability by the human body [9]. When peptides chelate with metal ions, the resulting complexes are more stable in the digestive system and exhibit higher bioavailability [10,11]. Furthermore, research indicates that peptide–metal chelates may possess the ability to cross the blood–brain barrier, thereby allowing for more direct action on targeted regions [12]. With regard to stability, the peptide coordinates with zinc ions via carboxyl oxygen, carbonyl oxygen, and amino nitrogen atoms to establish a robust chelation architecture, which allows the chelate to retain exceptional stability in simulated gastrointestinal environments, markedly surpassing that of zinc sulfate and zinc gluconate [13]. In terms of low toxicity, the peptide–zinc chelate exhibits higher cell viability and a lower risk of adverse reactions in vivo [14]. Zinc is an essential trace element in humans. The brain exhibits particularly high levels of zinc ions, which are thought to play a significant role in cognitive processes, including memory and related neural activities [15]. Deer brain has long been used in traditional medicine to enhance memory and improve sleep. Given that zinc ions are known to be associated with memory function, we hypothesize that peptide extracts from deer brain, when chelated with zinc ions, may offer a more effective treatment for memory impairment caused by insomnia.

Although research on peptide chelates has been fairly extensive, no chelation system using deer brain as the peptide source has been reported to date; this work therefore represents the first attempt in this regard. In traditional medicine, deer brain has long been regarded as a remedy for calming the mind, tranquilizing the spirit, and improving brain function. Ancient herbal classics documented its use in treating insomnia and mental restlessness. Owing to its scarcity, it was historically a precious medicinal substance accessible only to the nobility. By integrating the traditional medicinal value of deer brain with modern peptide chelation technology, we expect that the prepared deer brain peptides and their zinc chelates will exhibit superior bioactivity in ameliorating memory impairment induced by insomnia. This will provide experimental evidence for the development of new health supplements, foods, or drugs.

In this study, deer brain peptides (DBPPs) were extracted via enzymatic hydrolysis and subsequently chelated with Zn^2+^ to form zinc-chelated deer brain peptides (Zn-DBPP). The preparation process will be optimized using single-factor experiments and response surface methodology. The resulting DBPP and Zn-DBPP will be characterized by Fourier transform infrared spectroscopy (FTIR), ultraviolet–visible absorption spectroscopy (UV–Vis), amino acid content analysis, and scanning electron microscopy (SEM). For in vivo evaluation, a mouse model of insomnia will be induced using *p*-chlorophenylalanine (PCPA). The therapeutic effects and underlying mechanisms of DBPP and Zn-DBPP will be investigated through pentobarbital sodium assays, Morris water maze tests, atomic absorption spectrometry, hematoxylin–eosin (HE) staining, Nissl staining, immunofluorescence staining, and Western blot (WB) analysis.

## 2. Materials and Methods

### 2.1. Raw Materials and Reagents

Sika deer (Cervus nippon) brain samples were collected from Shuangyang District, Changchun City, China. Papain (800 U/g), Neutral Protease (100 U/g), Alkaline Protease (200 U/g), Trypsin (250 U/g), and Flavorzyme (20 U/g), as well as sodium hydroxide pellets, were purchased from Shanghai Yuanye Bio-Technology Co., Ltd., China. Ammonium chloride buffer, 4-chlorophenylalanine, 7,8-dihydroxyflavone, ANA-12, zinc sulfate, concentrated sulfuric acid, and gum arabic were purchased from Shanghai Macklin Biochemical Co., Ltd., China. DPPH radical scavenging activity assay kit and ABTS assay kit were purchased from Shanghai Jianglai Biotechnology Co., Ltd., China. Prestained Protein Ladder (10–180 kDa), RIPA Lysis Buffer, PMSF, protein buffer, 8% gel kit, and 15% gel kit were purchased from Biosharp, China. Electrophoresis buffer, electrotransfer buffer, protein-free rapid blocking solution, goat anti-rabbit IgG, anti-actin antibody, prestained protein ladder (2–40 kDa), and prestained protein ladder (10–180 kDa) were purchased from Wuhan Servicebio Technology Co., Ltd., China. Anti-BDNF antibody, anti-SYN antibody, anti-Trkb antibody, and anti-PSD95 antibody were purchased from HUABIO, China. Specific pathogen-free (SPF) KM mice were purchased from Changchun Yisi Experimental Animal Technology Co., Ltd., China.

### 2.2. Enzymatic Treatment of Deer Brain

Wash the deer brain, remove the meninges, and make it into a homogenate (the mass ratio of deer brain to deionized water is 1:4). Take 10 g of the homogenate and place it in a centrifuge tube. After adjusting the pH to an appropriate value, add an appropriate amount of enzyme preparations for enzymatic treatment. After the reaction is completed, heat it in a water bath at 90 °C for 10 min to inactivate the enzymes. Let it cool to room temperature and filter out the residues. Centrifuge the filtrate at 16,800× *g* at 4 °C for 10 min, and take the supernatant as the enzymatic product [16]. To reduce errors caused by individual differences and batch-to-batch variations in protein content during deer brain peptide preparation, all deer brains were processed together for homogenization. Three independent replicates were performed for each experiment.

### 2.3. Screening of Proteases

According to the hydrolysis conditions of each enzyme, five different proteases (neutral protease, trypsin, papain, alkaline protease, and flavor enzyme) were selected to carry out enzymatic hydrolysis on the deer brain. Among them, the alkaline protease had a hydrolysis pH of 10, while the pH of the other enzymes was 7. The ratio of substrate to liquid was 1/4 for all substrates, and the enzymatic hydrolysis was carried out at 45 °C for 4 h. The enzyme dosage was 6000 U/g for all.

### 2.4. Single-Factor Experiment

Alkaline protease was selected as the enzyme preparation. Under the condition of a solid–liquid ratio of 1/4, single-factor experiments were conducted on the enzymatic hydrolysis temperature, pH, enzyme dosage, and enzymatic hydrolysis time to screen out the optimal enzymatic hydrolysis conditions. The specific experimental steps are as follows.

#### 2.4.1. Enzymatic Hydrolysis Temperature

Under the action of alkaline protease, with a fixed enzymatic hydrolysis time of 4 h, an enzyme dosage of 6000 U/g, and a pH of 10, the effects of enzymatic hydrolysis temperatures of 40 °C, 45 °C, 50 °C, 55 °C and 60 °C on the hydrolysis degree of deer brain were investigated.

#### 2.4.2. pH

Under optimal temperature conditions, with a fixed enzymatic hydrolysis time of 4 h and an enzyme dosage of 6000 U/g, the effects of pH values of 9, 9.5, 10, 10.5, 11, and 11.5 on the degree of hydrolysis of deer brain were investigated.

#### 2.4.3. Enzyme Dosage

Under the optimal temperature and pH conditions, with a fixed enzymatic hydrolysis time of 4 h, the effects of enzyme dosages of 2000, 4000, 6000, 8000, 10,000, and 12,000 U/g on the hydrolysis degree of deer brain were investigated.

#### 2.4.4. Enzymatic Hydrolysis Time

Under the optimal temperature, pH and enzyme dosage conditions, the effects of enzymatic hydrolysis times of 2, 3, 4, 5, and 6 h on the hydrolysis degree of deer brain were investigated.

### 2.5. Experimental Design of Response Surface

Based on single-factor experiments, the factors selected as influencing factors were enzymatic hydrolysis temperature (A), pH (B), enzyme concentration (C), and enzymatic hydrolysis time (D), and the hydrolysis degree (DH) was taken as the response value. The response surface methodology with three levels and four factors was used to optimize the enzymatic hydrolysis process of deer brain.

### 2.6. Determination of Hydrolysis Rate (DH)

The determination process is carried out according to the pH-STAT method [17,18]. During the enzymatic hydrolysis process, 0.1 moles/liter of sodium hydroxide is used to maintain a constant pH value, and the volume (in milliliters) of the consumed sodium hydroxide is recorded. The calculation formula is as follows:

DH = (Vb × cb)/(α × mp × htot) × 100%

where Vb denotes the volume of alkali solution consumed during titration (mL). cb denotes the concentration of the alkali solution (mol/L). α denotes the average degree of dissociation of amino acids, where α = 10^(pH − pK)/[1 + 10^(pH − pK)] and pK = 7.8 + [(298 − T)/(298 × T)]. mp denotes the mass of protein in the substrate (g). htot denotes the total number of peptide bonds in the substrate protein (mmol/g).

### 2.7. Preparation of DBPP Chelated Zinc

Dissolve the freeze-dried peptides in deionized water, add an appropriate amount of zinc sulfate, and then incubate the mixture in a water bath under suitable conditions of pH, temperature, and time to allow the chelation reaction to proceed. After the reaction was complete, anhydrous ethanol was added to the reaction mixture to precipitate the chelated product. The resulting suspension was then centrifuged at 11,200× *g* for 10 min. The supernatant was discarded, and the precipitate was collected and lyophilized to obtain the final chelate [19]. According to the above procedure and the single-factor approach in Section 2.4, the influences of pH, mass ratio of ZnSO_4_ to DBPP (mZnSO_4_:mDBPP), temperature, and time were examined in turn via single-factor experiments.

### 2.8. Zn-DBPP Response Surface Design

Based on the single-factor experimental results, pH (A), temperature (B), and time (C) were selected as the influencing factors, and the zinc content in the chelated zinc product (mg/g) was taken as the response value. A three-factor response surface methodology (RSM) was then employed to optimize the chelation process of deer brain peptide.

### 2.9. Determination of Zinc Content

Take 2 g of Zn-DBPP and dissolve it in 100 mL of deionized water. Take 20 mL of this solution, add 5 mL of NH_3_-NH_4_Cl buffer solution and 2 drops of Eriochrome Black T indicator, and mix with 0.01 mol/L EDTA standard solution. Titrate until the color changes from purple to blue [20]. The Zn content in the deer brain peptide chelate is calculated using the following formula: X (mg/g) = M × c × V × 5/m. Here, X is the zinc content in the chelate (mass fraction), M is the relative atomic mass of zinc, c is the concentration of the standard EDTA titrant (mol/L), V is the volume of the standard EDTA titrant consumed (mL), and m is the mass of the chelate sample weighed (g) [19].

### 2.10. Antioxidant Capacity Determination

#### 2.10.1. DDPH Clearance Rate Determination

The measurement is performed using a Jianglai biological reagent kit. Specifically, 150 µL of sample solutions at concentrations of 2, 4, 6, 8, and 10 mg/mL are each mixed with 150 µL of DPPH working solution, and the mixtures are kept in the dark for 30 min. Then, the absorbance (A) at 517 nm is measured using a UV–visible spectrophotometer, USA. DDPH clearance rate (%) = [1 − (A1 − A2)/A0] × 100% [21]. Where A1 is the absorbance of 150 μL of sample mixed with 150 μL of DPPH working solution, A2 is the absorbance of 150 μL of sample mixed with 150 μL of 80% methanol, and A0 is the absorbance of 150 μL of 80% methanol mixed with 150 μL of DPPH working solution.

#### 2.10.2. Determination of ABTS Clearance Rate

The measurement was performed using the ABTS assay kit Hualian Biotechnology, China. Briefly, 10 µL of sample at concentrations of 2, 4, 6, 8, and 10 mg/mL was mixed with 190 µL of the working solution. The reaction was incubated in the dark for 6 min, and the absorbance was measured at 734 nm. ABTS free radical scavenging rate (%) = (A0 − A1) ÷ A0 × 100% [22]. Where A_1_ represents the absorbance value of 10 μL of sample mixed with 190 μL of working solution, and A_0_ denotes that of 10 μL of extract mixed with 190 μL of working solution.

### 2.11. Characterization and Analysis of Deer Brain Peptides and Their Complexes

#### 2.11.1. Ultraviolet Spectroscopy Determination

The deer brain peptide and the zinc-chelated deer brain peptide were prepared into a 1 milligram per milliliter aqueous solution. Subsequently, measurements were conducted using a UV–visible spectrophotometer within the range of 200–400 nanometers, and a deionized water solution was used as the blank for calibration [23].

#### 2.11.2. Infrared Spectroscopy Determination

We separately weighed 2 mg of deer brain peptides and deer brain peptides chelated with zinc, and mixed them with 200 mg of dry potassium bromide. They were ground into a fine powder and pressed into tablets. The samples were scanned using a Fourier transform infrared spectrometer, USA in the wavenumber range of 4000–400 cm^−1^ [19].

#### 2.11.3. Amino Acid Composition Analysis

The deer brain peptide and the deer brain peptide–zinc chelate samples were placed in a sealed digestion container. Subsequently, under vacuum and nitrogen protection, they were subjected to a 22 h hydrolysis treatment at 110 °C using 6 molar/liter concentration hydrochloric acid. After the hydrolysis was completed, the residual hydrochloric acid was removed by distillation, and the resulting solution was diluted to an appropriate concentration. Then, the solution was filtered through a water-based filtration membrane with a pore size of 0.22 micrometers. Finally, the amino acid components in the filtrate were analyzed in detail using an amino acid analyzer, USA [24].

#### 2.11.4. Scanning Electron Microscopy (SEM) Measurement

The powders of deer brain peptides and deer brain peptides combined with zinc were gold-coated, and then their microstructures under different magnification levels were observed using a scanning electron microscope (SEM), USA [25].

### 2.12. Model Construction

After one week of adaptive feeding, except for the control group, the other groups of mice were intraperitoneally injected with *p*-Chlorophenylalanine (PCPA) suspension (450 mg/kg, dissolved in Arabic gum, pH 7–8) every day for 3 consecutive days. During the modeling period, the behavior of the mice was observed. Compared with the blank group, the mice in the other groups showed typical insomnia symptoms such as disrupted circadian rhythm, increased daytime activity, disheveled hair, enhanced aggression, weight loss, and grayish-white feces, indicating that the insomnia model was successfully established. 72 h after the last injection of PCPA, the sleep latency and sleep time were recorded using the pentobarbital sodium experiment to further verify the success of the model [26].

### 2.13. Experimental Animal Grouping and Drug Administration

#### 2.13.1. Grouping of Experimental Animals for Drug Efficacy Tests

Purchased 60 specific pathogen-free (SPF) Kunming (KM) mice from Changchun Yisi Company, China, male, 4 weeks of age, each weighing 20 ± 5 g. After 7 days of acclimatization, they were randomly divided into six groups (*n* = 10 per group, The sample size was determined based on a review of extensive literature reports.): a control group (CON), a model group (MOD), a positive drug group (POS) treated with Zhennaoning Capsules [27] (as a positive control), a deer brain peptide group (DBPP), a low-dose zinc–deer brain peptide group (L-Zn-DBPP), and a high-dose zinc–deer brain peptide group (H-Zn-DBPP). Drug administration was initiated after modeling. The POS group received Zhennaoning Capsules (200 mg/kg) by gavage, the DBPP group received DBPP (200 mg/kg) by gavage, the L-Zn-DBPP group received Zn-DBPP (100 mg/kg) by gavage, and the H-Zn-DBPP group received Zn-DBPP (200 mg/kg) by gavage. All administrations were performed once daily for 14 consecutive days. 

#### 2.13.2. Experimental Animal Grouping for the Mechanism Study

Purchased 50 specific pathogen-free (SPF) Kunming (KM) mice from Changchun Yisi Company, male, 4 weeks of age, each weighing 20 ± 5 g. After 7 days of acclimatization, they were randomly divided into five groups (*n* = 10 per group): control (CON) group, model (MOD) group, (H-Zn-DBPP) group, Inhibitor (INH) group, and Agonist (AGO) group. The H-Zn-DBPP group received Zn-DBPP (200 mg/kg) via intragastric administration for 14 days after successful modeling (which was performed following 7 days of acclimatization and 3 days of model induction). The INH group received the TrkB receptor antagonist Ana-12 (0.5 mg/kg) via intraperitoneal injection during the last 7 days of the experimental period. The AGO group received the agonist 7,8-dihydroxyflavone (5 mg/kg) via intraperitoneal injection during the last 7 days of the experimental period. All treatments were administered once daily. 

#### 2.13.3. Housing Conditions and Humane Measures

All experimental mice were housed in an SPF-grade barrier facility under controlled environmental conditions, with ambient temperature maintained at 20–26 °C, relative humidity at 30–70%, and a 12 h light/12 h dark cycle. The mice had ad libitum access to a complete pelleted diet and drinking water. Corncob or aspen shavings were used as bedding, which was changed once per week, and no more than five mice were housed per cage.

All invasive procedures were performed under aseptic conditions. The number of manipulations and the duration of each session were minimized throughout the experiment. Animals were monitored at least once daily, and when necessary, the frequency was increased to twice daily. The experiment was immediately terminated and euthanasia was performed when any of the following criteria were met: body weight loss exceeding 20% of the initial body weight; complete loss of appetite for 24 h, or food intake below 50% of the normal level for 3 consecutive days; signs of severe pain, distress, or irreversible injury; presentation of a moribund state, inability to feed or drink independently, or inability to make normal postural adjustments.

If unexpected adverse events occur during the experiment, the relevant procedures are immediately ceased, and the animal’s condition is assessed by a veterinarian or the principal investigator. For reversible adverse events, timely symptomatic treatment is administered. For severe adverse events that are irreversible or cannot be alleviated, euthanasia is performed immediately. At the end of the experiment, mice are euthanized by CO_2_ inhalation.

#### 2.13.4. Exclusion Criteria for Experimental Animals and Rationale for Sample Size

Animals showing signs of diarrhea, hair loss, or spontaneous tumors during the acclimation period (*n* = 7). Animals that died or experienced severe complications during administration or modeling procedures (*n* = 11). Animals found to have organic lesions in major organs not caused by the experimental procedures at necropsy (*n* = 3). Animals with test data that are extreme outliers exceeding the mean ± 3 standard deviations (*n* = 10). Animals excluded from the experimental data due to the above reasons: In the pharmacodynamic experiment, 4 mice in the MOD group were not included. In the mechanism experiment, 3 mice in the MOD group were not included. In the pharmacodynamic experiment, 2 mice in the CON group were not included. In the mechanism experiment, 3 mice in the CON group were not included. In the pharmacodynamic experiment, 3 mice in the H-Zn-DBPP group were not included. In the mechanism experiment, 3 mice in the H-Zn-DBPP group were not included. In the POS group, 3 mice were not included. In the DBPP group, 2 mice were not included. In the L-Zn-DBPP group, 2 mice were not included. In the AGO group, 2 mice were not included. In the INH group, 4 mice were not included.

Due to the exclusion of certain mice from some groups for the reasons described above, only 6 animals remained per group; therefore, we used a sample size of *n* = 6 for all behavioral tests (including body weight, brain zinc content, and sleep experiments). Given the stringent requirements for sample preparation quality in immunofluorescence, hematoxylin–eosin (H&E) staining, and Nissl staining (e.g., absence of section folds and staining inhomogeneity), we performed these experiments with *n* = 3. Western blot analysis was also conducted with *n* = 3, which met the criterion of three independent replicates. Although the final sample sizes (*n* = 6 or *n* = 3) were lower than the initial values, they still fell within the acceptable range for statistical analysis.

#### 2.13.5. Blinding Procedure

During the randomization and animal dosing phases, all personnel were aware of the group assignments. In the experimental testing phase, all samples were recoded with numeric labels so that the evaluators could see only the sample numbers and were blinded to the corresponding group allocations. The group assignments were revealed only after the data analysis was completed.

### 2.14. Experiment on Inducing Sleep with Pentobarbital Sodium

On the 13th day, after the mice received a 30 min treatment, a pentobarbital sodium-induced sleep test was conducted immediately [28]. Each mouse was administered pentobarbital sodium (55 mg/kg), and it was observed that the sleep onset time was 60 min. When the mice lost the righting reflex for more than 60 s, they were considered to be in a sleep state. The sleep latency was recorded as the time from the injection of pentobarbital sodium until the righting reflex was lost. The duration of sleep was measured as the elapsed time between the onset and termination of the loss of righting reflex (LORR).

### 2.15. Determination of Zinc Content in the Brain of Mice

On days 7 and 14 after administration, the brain zinc levels in the H-Zn-DBPP group were recorded and compared with those in the normal group. Subsequently, by observing the changes in zinc levels among the three groups, we assessed whether H-Zn-DBPP could elevate brain zinc levels in mice.

### 2.16. Morris Water Maze Experiment

The Morris water maze test was conducted over six days: the first four days for training, day 5 for the place navigation test (hidden platform test), and day 6 for the spatial probe test.

Training: A visible platform was marked and kept above the water surface, and the water was maintained clear. Mice were placed into the pool from a fixed entry point. If a mouse found the platform within 90 s, it was allowed to remain on it for 15 s. If it failed to find the platform, it was guided to the platform and allowed to stay for 15 s. Training was performed twice per day.

Place navigation test (day 5): Diatomaceous earth was added to make the water opaque, and the platform marker was removed so that the platform was submerged below the water surface. Each mouse was released from the same fixed entry point used during training. The time taken to find the platform (escape latency) and the swimming distance from entry to platform were recorded within a 90 s limit.

Spatial probe test (day 6): The platform was removed, while all other conditions remained unchanged. The number of times each mouse crossed the original platform location was recorded within 90 s [29].

### 2.17. HE Staining and Nissl Staining

HE staining procedure: The sections were sequentially dewaxed and rehydrated as follows: immersion in xylene I and II for 20 min each, followed by absolute ethanol I and II for 5 min each, then 75% ethanol for 5 min, and finally rinsed with distilled water. The sections were stained with hematoxylin solution for 5 min, washed with water, treated with bluing solution for differentiation, and rinsed again with water. Subsequently, the sections were dehydrated through graded ethanol: 85% ethanol for 5 min, 95% ethanol for 5 min, and then stained with eosin solution for 5 min. After that, the sections were dehydrated with absolute ethanol I, II, and III for 5 min each, cleared with xylene I and II for 5 min each, and finally mounted with neutral gum. Microscopic observation and image acquisition were performed for analysis [30].

Nissl staining: Brain tissue paraffin sections were routinely deparaffinized, rehydrated to distilled water, and then immersed in 1% thionin staining solution. The sections were incubated in a water bath for 5 min, followed by a 2 min rinse in distilled water. Excess stain was removed by differentiation in 90% ethanol. Subsequently, the sections were dehydrated through a graded ethanol series, cleared in xylene, and mounted with neutral gum. Finally, neuronal morphology in the CA1 region of the hippocampus was observed under a light microscope [31].

### 2.18. Immunofluorescence Staining

Paraffin-embedded brain tissue sections from each group of rats were routinely deparaffinized and rehydrated in distilled water. Antigen retrieval was performed using citrate buffer (pH 6.0), followed by washing with PBS. After drawing a hydrophobic barrier around the sections with an immunohistochemistry pen, the sections were sequentially treated with an autofluorescence quenching agent and then blocked with BSA. The blocking solution was discarded, and the sections were incubated with primary antibodies against BDNF, TrkB, PSD-95, and SYN (all at a dilution of 1:100) in a humidified chamber at 4 °C overnight. On the next day, the sections were washed with PBS and incubated with the corresponding fluorescent secondary antibodies (diluted 1:300) at room temperature in the dark for 50 min. After washing with PBS, the sections were stained with DAPI solution at room temperature in the dark for 10 min. Following another wash with PBS, the sections were mounted with an anti-fade mounting medium. Finally, the sections were observed and photographed under a fluorescence microscope, and the average fluorescence intensity for each marker was analyzed using SlideViewer 2.6 and Fiji 2.14.0 software.

### 2.19. Western Blotting (WB) Experiment

After the samples were extracted and quantified using RIPA lysis buffer (RIPA) (containing protease/phosphatase inhibitors), they were mixed with the loading buffer and heated at 100 °C for 10 min. Each well was loaded with 4–5 μg of total protein. First, an 80 V stacking gel was used, followed by a 120 V separating gel for electrophoresis. The membrane was transferred using the wet transfer method on a Polyvinylidene Fluoride Membrane, with a transfer solution containing 20% anhydrous ethanol. The transfer was performed in an ice bath at 300 mA for 90–120 min. Subsequently, the membrane was blocked with rapid blocking solution for 30 minutes at room temperature, and then incubated with the primary antibody overnight at 4°C. The membrane was washed and then incubated with Horseradish Peroxidase (HRP) secondary antibody at room temperature for 1 h. Finally, Enhanced Chemiluminescence (ECL) imaging was used for detection.

### 2.20. Statistical Analysis

All quantitative data are presented as the mean ± SD from a minimum of three independent replicates. Statistical comparisons were performed using Student’s *t*-test or one-way ANOVA, as appropriate. For multiple group comparisons, one-way ANOVA accompanied by Tukey’s post hoc test was applied. Analyses were carried out using SPSS 25.0 (SPSS Inc., Chicago, IL, USA), GraphPad Prism v8.0 (GraphPad Software Inc., La Jolla, CA, USA), and FlowJo V10. Data were considered statistically significant when *p* < 0.05.

## 3. Results

### 3.1. Results of Process Optimization of DBPP

#### 3.1.1. Results of the Single-Factor Experiment

**Figure 1:**
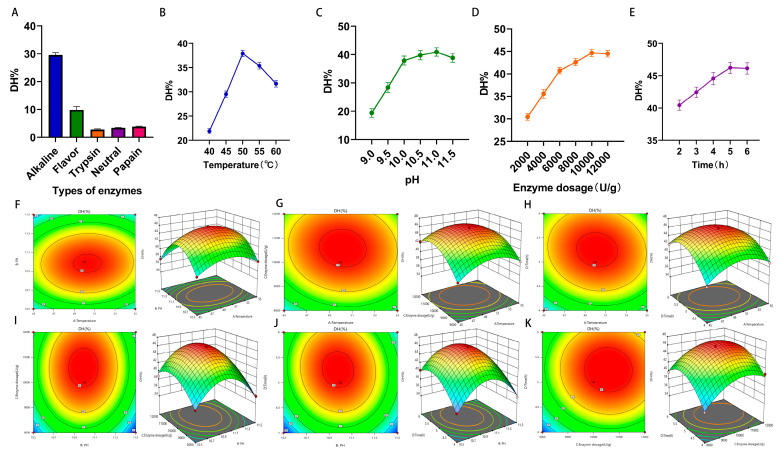
(**A**) Single-factor experimental results for protease type; (**B**) temperature; (**C**) pH; (**D**) enzyme dosage; (**E**) time. (**F**–**K**) Response surface plots.

The single-factor experiment in Figure 1A indicated that alkaline protease was the most suitable enzyme, yielding a degree of hydrolysis (DH = 29.54%). Further investigations into the effects of temperature, pH, enzyme dosage, and hydrolysis time on the DH of deer brain revealed the following: As shown in Figure 1B, the DH initially increased with rising temperature, peaked at 50 °C (DH = 37.99%), and then declined. The optimal temperature range was determined to be 45–55 °C. Figure 1C demonstrates that the DH increased with increasing pH, reaching its highest value at pH 11 (DH = 40.65%), with an optimal range of 10.5–11.5. According to Figure 1D, the DH increased with enzyme dosage, reaching a maximum at 10,000 U/g (DH = 44.52%) before plateauing; the optimal dosage range was 8000–12,000 U/g. Figure 1E shows that the DH increased with prolonged hydrolysis time, peaking at 5 h (DH = 46.23%) and then slightly decreasing, with an optimal time range of 4–6 h.

#### 3.1.2. Results of DBPP Response Surface

The results of the response surface experiment and the results of the variance analysis are presented in Table 1 and Table 2, respectively.

A multiple regression analysis was conducted on the data, and the following regression equation was obtained: Degree of hydrolysis DH (%) = 46.19 + 0.3017 ∗ A − 0.3358 ∗ B + 1.80 ∗ C + 1.73 ∗ D + 0.2375 ∗ AB − 0.4075 ∗ AC − 0.5250 ∗ AD + 0.1375 ∗ BC − 0.5825 ∗ BD − 0.2225 ∗ CD − 2.68 ∗ A^2^ − 5.55 ∗ B^2^ − 3.04 ∗ C^2^ − 3.35 ∗ D^2^.

As shown in Table 2 and Table 3, the correlation coefficient R^2^ of the regression equation is 0.9898, the F-value of the model is 83.07, and the *p*-value is less than 0.0001, indicating that the model is highly significant. The *p*-value for the lack of fit is 0.0810, which is not significant, suggesting a high goodness of fit of the regression equation. Among the factors, the linear terms C and D, as well as the quadratic terms A^2^, B^2^, C^2^, and D^2^, have a highly significant effect on the degree of hydrolysis of deer brain by alkaline protease (*p* < 0.01). The linear terms A and B, along with the interaction terms AD and BD, have a significant effect (*p* < 0.05), whereas the interaction terms AB, AC, BC, and CD are not significant (*p* > 0.05).

#### 3.1.3. DBPP Response Surface Analysis

Response surface plots provide a visual representation of how interactions between different factors influence the response variable. Generally, the steeper the surface along a given factor’s direction, the more substantial that factor’s effect; conversely, a flatter surface indicates a weaker effect. The shape of the contour lines further reveals the nature of interactions: elliptical contours suggest a strong interaction between two factors, whereas circular contours imply a weak one. The response surface trends for all factor combinations are illustrated in Figure 1F–K. Specifically, Figure 1H and 1J show elliptical contour patterns for the factor pairs AD and BD, respectively, indicating pronounced interactions between these pairs. This finding is consistent with the results of the analysis of variance ANOVA and confirms that the interactions among the experimental parameters are nonlinear. Notably, the contour lines for BD in Figure 1J exhibit the highest degree of ellipticity, underscoring that pH and reaction time have a significant impact on the degree of hydrolysis of deer brain. Therefore, manipulating these two parameters offers an effective means of controlling the enzymatic hydrolysis process.

#### 3.1.4. Prediction and Verification of Optimal Enzymatic Hydrolysis Conditions for DBPP

Response surface analysis determined the optimal enzymatic hydrolysis conditions as a temperature of 49.832 °C, pH of 11.052, enzyme dosage of 10,457.201 U/g, and reaction time of 5.270 h, achieving a hydrolysis rate of 46.542%. To enhance practical applicability and further verify the predictive accuracy of the response surface methodology, the optimal conditions were adjusted to 50 °C, pH 11.0, an enzyme dosage of 10,460 U/g, and a reaction time of 5.3 h. Under these modified conditions, the experimental enzymatic hydrolysis rate reached 46.489%, which was not significantly different from the predicted value of 46.542% (*p* > 0.05) within the acceptable experimental error range. These results confirm that response surface methodology is an accurate and reliable tool for optimizing the enzymatic hydrolysis conditions of alkaline protease.

### 3.2. Results of Process Optimization of Zn-DBPP

#### 3.2.1. Results of Zn-DBPP Single-Factor Experiment

**Figure 2:**
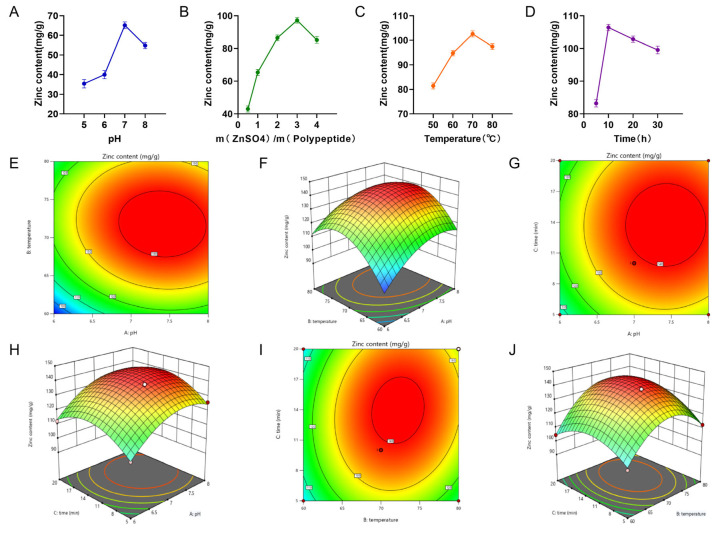
(**A**) Effect of single-factor pH on zinc content. (**B**) Effect of single-factor mDBPP:mZn on zinc content. (**C**) Effect of single factor temperature on zinc content. (**D**) Effect of single factor time on zinc content. (**E**–**J**) Planar and three-dimensional diagrams of the interaction between two factors in the response surface pH, temperature and time.

**Figure 3:**
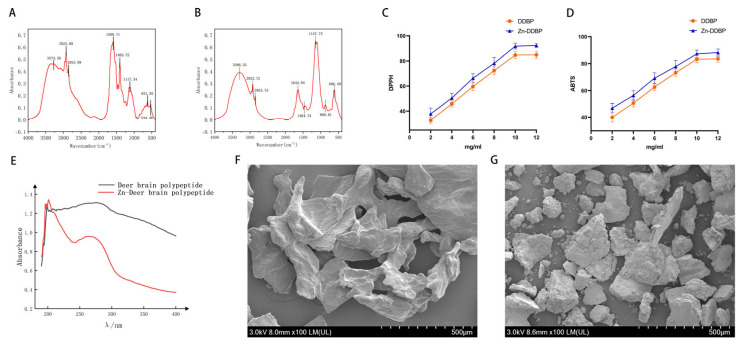
(**A**) FTIR spectrum of DBPP. (**B**) FTIR spectrum of Zn-DBPP. (**C**) DPPH radical scavenging activity. (**D**) ABTS radical scavenging activity. (**E**) UV spectra of DBPP and Zn-DBPP. (**F**) SEM image of DBPP. (**G**) SEM image of Zn-DBPP.

As shown in Figure 2A, the highest chelated zinc content was 65.03 mg/g at pH 7. As shown in Figure 2B, the highest chelated zinc content was 97.32 mg/g at a DBPP:ZnSO_4_ mass ratio of 1:3. As shown in Figure 3C, the highest chelated zinc content was 102.74 mg/g at 70 °C. As shown in Figure 3D, the highest chelated zinc content was 106.35 mg/g at a chelation time of 10 min. Based on all the above factors, the optimal range of chelation conditions was determined, providing a basis for subsequent response surface methodology to optimize the preparation process of Zn-DBPP.

#### 3.2.2. Results of Zn-DBPP Response Surface Experiment

The results of the response surface experiments for the chelation of deer brain peptides and the results of the variance analysis are presented in Table 3 and Table 4, respectively.

The data were subjected to multiple regression analysis, and the following regression equation was obtained: Zinc content (mg/g) = 140.93 + 9.16 ∗ A + 7.42 ∗ B + 3.51 ∗ C − 1.98 ∗ AB + 0.3067 ∗ AC + 3.32 ∗ BC − 10.84 ∗ A^2^ − 18.41 ∗ B^2^ − 10.96 ∗ C^2^. As can be seen from Table 4 and Table 5, the correlation coefficient R^2^ of the regression equation is 0.943. The model exhibits an F-value of 97.45 and a *p*-value of less than 0.0001, indicating that the model is extremely significant. The *p*-value for the lack of fit is 0.5071, which is not significant, suggesting a high degree of fit for the regression equation. Among all factor levels, the linear terms A, B, and C, as well as the quadratic terms A^2^, B^2^, and C^2^, had extremely significant effects on the zinc content of the chelate (*p* < 0.01). The interaction term BC had a significant effect (*p* < 0.05), while AB and AC showed no significant effects (*p* > 0.05).

#### 3.2.3. Response Surface Analysis

Response surface plots provide a visual representation of how interactions between different factors influence the response variable. Generally, the steeper the surface along a given factor’s direction, the more substantial that factor’s effect; conversely, a flatter surface indicates a weaker effect. The shape of the contour lines further reveals the nature of interactions: elliptical contours suggest a strong interaction between two factors, whereas circular contours imply a weak one. The influence trends of each factor on the response surface are shown in Figure 2E–J. As seen in Figure 2F,J, the contour lines for AB and BC are elliptical, indicating strong interactions between these factor pairs, which is consistent with the ANOVA results. This suggests that the mutual influences among parameters in this experiment are nonlinear. Notably, the contour ellipse for BC in Figure 2J exhibits the highest degree of elongation, indicating that temperature and time significantly affect the zinc content in the chelate. Variations in these parameters can effectively control the progression of the chelation reaction.

#### 3.2.4. Prediction and Validation of Optimal Chelation Conditions for Zn-DBPP

After optimizing the model parameters, the highest Zn-DBPP zinc content was achieved under the following conditions: pH 7.531, temperature 72.803 °C, reaction time 13.628 min, and a substrate-to-zinc sulfate ratio of 1:3. To account for practical applicability and to validate the accuracy of the response surface methodology, the reaction conditions were adjusted to pH 7.5, temperature 73 °C, time 13.6 min, with the same substrate-to-zinc sulfate ratio of 1:3. Under these conditions, the experimentally determined zinc content was 143.37 mg/g. Within the permissible margin of error, this experimental value showed no significant difference from the theoretically predicted value of 143.518 mg/g (*p* < 0.05). These findings collectively demonstrate that the chelation conditions established via response surface methodology are both accurate and reliable.

### 3.3. Results of Characterization of DBPP and Zn-DBPP

#### 3.3.1. Results of Amino Acid Analysis of DBPP and Zn-DBPP

Both DBPP and Zn-DBPP contain 17 kinds of amino acids and all the essential amino acids for the human body. Per 100 g, DBPP contains 67.93 g of total amino acids, whereas Zn-DBPP contains 53.26 g. Among them, the proportions of glutamic acid, aspartic acid, leucine, lysine, etc., are relatively high. After chelation, the proportion of glutamic acid increases from 15.43% to 16.94%. The high content of glutamic acid combined with zinc ions helps maintain the stability of the nervous system, providing a basis for the therapeutic effect in animal experiments. However, we found that the amino acid content of Zn-DBPP was lower than that of DBPP. This may be because free oligopeptides that did not participate in chelation were discarded during the re-chelation process. As a result, the total peptide abundance in the final lyophilized chelate was lower than that in the original peptide fraction, leading to a decrease in the measured amino acid content.

#### 3.3.2. Result of Fourier Transform Infrared Spectroscopy (FTIR) of DBPP and Zn-DBPP

Comparison of the Fourier transform infrared (FTIR) spectra of DBPP and Zn-DBPP in Figure 3A,B reveals that Zn^2+^ chelates primarily through the amide groups of the peptide. Upon chelation, the N–H stretching band shifts from 3274 cm^−1^ to 3286 cm^−1^, indicating the involvement of the amide nitrogen atoms [32]. Meanwhile, a new absorption band appears at 1644 cm^−1^ in Zn-DBPP, attributed to the amide I (C=O stretching) vibration, which is absent in DBPP, suggesting the participation of carbonyl oxygen atoms in the chelation [33]. The disappearance of the band at 1403 cm^−1^ and the appearance of a band at 1147 cm^−1^ further confirm the formation of stable chelates between Zn^2+^ and the N/O donors of the peptide backbone [34].

#### 3.3.3. Results of DPPH and ABTS Clearance Rate Determination

Figure 3C demonstrates that at a concentration of 10 mg/mL, both DBPP and Zn-DBPP exhibited their maximum DPPH radical scavenging activities, reaching 84.73% and 91.81%, respectively. Similarly, Figure 3D reveals that at the same concentration, the ABTS scavenging activities of DBPP and Zn-DBPP also peaked at 83.3% and 87.28%, respectively. Together, these results indicate that Zn-DBPP possesses significantly stronger antioxidant activity than DBPP, likely due to the enhanced antioxidative capacity conferred by chelation with zinc ions.

#### 3.3.4. Results of Ultraviolet (UV) Analysis of DBPP and Zn-DBPP

Based on the spectral data presented in Figure 3E, the chelation of Zn^2+^ with DBPP induces distinct changes: an increase in absorbance within the low-wavelength region (175–185 nm) and a pronounced decrease in the high-wavelength region (190–390 nm), with the hypochromic effect becoming more evident at longer wavelengths. These spectral features are indicative of a coordination interaction between Zn^2+^ and DBPP, rather than a simple physical mixture. Specifically, Zn^2+^ coordinates with electron-donating moieties in the peptide chain—including amino nitrogen, carboxyl oxygen, and carbonyl oxygen of the peptide bonds—thus leading to the formation of the Zn–DBPP complex.

#### 3.3.5. Result of Scanning Electron Microscopy (SEM) Analysis

**Figure 4:**
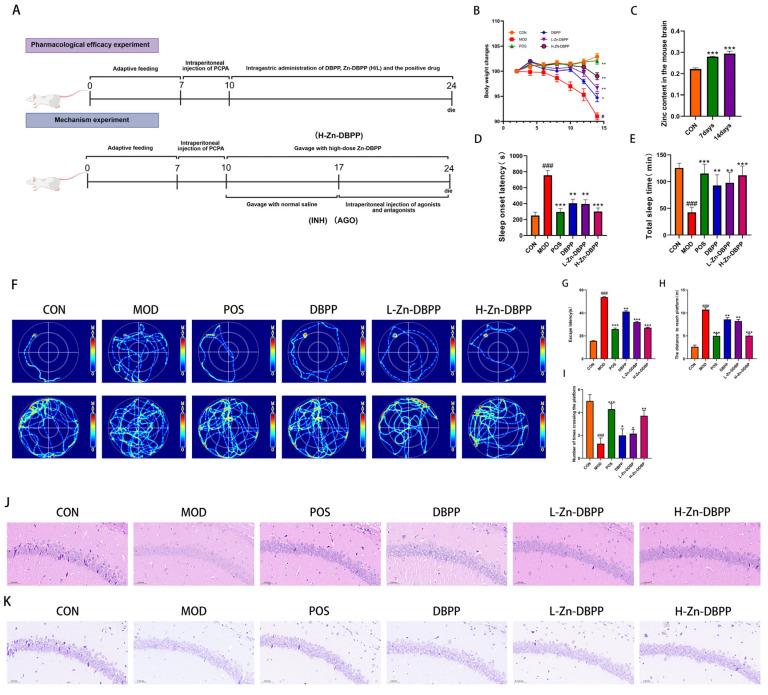
(**A**) Schematic diagram of the dosing regimens for pharmacodynamic and mechanistic experiments. (**B**) Body weight changes in animals (*n* = 6). (**C**) Zinc content in mouse brain (*n* = 6). (**D**) Sleep onset latency (*n* = 6). (**E**) Total sleep time(*n* = 6). (**F**) Morris water maze place navigation test and spatial probe test (*n* = 6). (**G**) Escape latency in the place navigation test (*n* = 6). (**H**) Distance to reach the platform (*n* = 6). (**I**) Number of platform crossings (*n* = 6). (**J**) H&E stain (*n* = 3). (**K**) Nissl stain (*n* = 3). (**B**,**D**,**E**,**G**,**H**,**I**) (# *p* < 0.05, ## *p* < 0.01, ### *p* < 0.001 vs. CON group. * *p* < 0.05, ** *p* < 0.01, *** *p* < 0.001 vs. MOD group). (**C**) (*** *p* < 0.001 vs. CON group).

As shown in Figure 4F,G, SEM revealed a smooth surface for DBPP. In contrast, Zn-DBPP showed a rough and cracked surface after Zn^2+^ chelation, likely resulting from the interaction between DBPP and Zn^2+^ that caused structural changes. SEM thus provided supplementary evidence for chelate formation.

### 3.4. Results of Body Weight Change

As shown in Figure 4B, the MOD group exhibited a significant reduction in body weight compared with the control group (*p* < 0.05). In contrast, treatment with H-Zn-DBPP led to a marked increase in body weight relative to the MOD group (*p* < 0.01), representing the greatest improvement among all treatment groups and reaching a level comparable to that of the positive control group.

### 3.5. Results of Atomic Content Quantification

As shown in Figure 4C, compared with the control group, the brain zinc content in the H-Zn-DBPP group was significantly increased on days 7 and 14 after administration (*p* < 0.001), indicating that Zn-DBPP treatment elevated brain zinc content in mice.

### 3.6. Results of Pentobarbital Sodium-Induced Sleep Experiment

The sedative and hypnotic effects of DBPP and Zn-DBPP were evaluated using the pentobarbital-induced sleep test. As shown in Figure 4D, the sleep latency in the MOD group was significantly longer than that in the control group (*p* < 0.001). Compared with the MOD group, treatment with DBPP and Zn-DBPP significantly shortened sleep latency, with the most pronounced effect observed in the H-Zn-DBPP group (*p* < 0.001). Figure 4E shows that the sleep duration in the MOD group was significantly shorter than that in the control group (*p* < 0.001). Relative to the MOD group, DBPP and Zn-DBPP treatments markedly prolonged sleep duration, and the H-Zn-DBPP group exhibited the greatest improvement (*p* < 0.001). These results indicate that the insomnia model was successfully established and that both DBPP and Zn-DBPP effectively alleviate PCPA-induced insomnia symptoms in mice.

### 3.7. Results of the Morris Water Maze Experiment

As shown in Figure 4F, mice in the MOD group exhibited significantly poorer performance in both the place navigation test and the spatial probe test of the Morris water maze compared with the control group. Administration of DBPP and Zn-DBPP significantly ameliorated the performance deficits in both tests relative to the MOD group. Figure 4G–I further demonstrated that DBPP and Zn-DBPP treatment significantly reduced escape latency and swimming distance during place navigation, and significantly increased the number of platform crossings in the spatial probe test, with the most pronounced improvement observed in the H-Zn-DBPP group.

### 3.8. Results of HE Staining and Nissl Staining

#### 3.8.1. Result of HE Staining

As shown in Figure 4J, H&E staining of the hippocampal CA1 region in the control group revealed a well-defined laminar structure, with densely and orderly arranged cells. The pyramidal cells exhibited intact morphology, and the neuronal count was within the normal range. In contrast, the hippocampal CA1 region of the model group displayed disorganized and disordered cell arrangement, accompanied by marked cell loss. Some nuclei appeared fragmented, the number of pyramidal cells was significantly reduced, and gliosis was observed. Compared with the MOD group, treatment with DBPP and Zn-DBPP ameliorated the pathological changes in the hippocampal CA1 region, with the most pronounced improvement seen in the H-Zn-DBPP group.

#### 3.8.2. Results of Nissl Staining

As shown in Figure 4K, the CA1 pyramidal cells in the CON group were densely packed, with abundant cytoplasm, and the neuroblasts exhibited prominent blue staining. In contrast, the CA1 neurons in the MOD group were loosely arranged, with obvious cell loss and disappearance of neuroblasts. Compared with the MOD group, treatment with DBPP and Zn-DBPP significantly ameliorated the pathological changes in the CA1 region, with the most marked improvement observed in the H-Zn-DBPP group.

### 3.9. Results of Immunofluorescence and Western Blot Experiments on the Efficacy and Mechanism of Zn-DBPP

#### 3.9.1. Results of Pharmacodynamic Experiment

**Figure 5:**
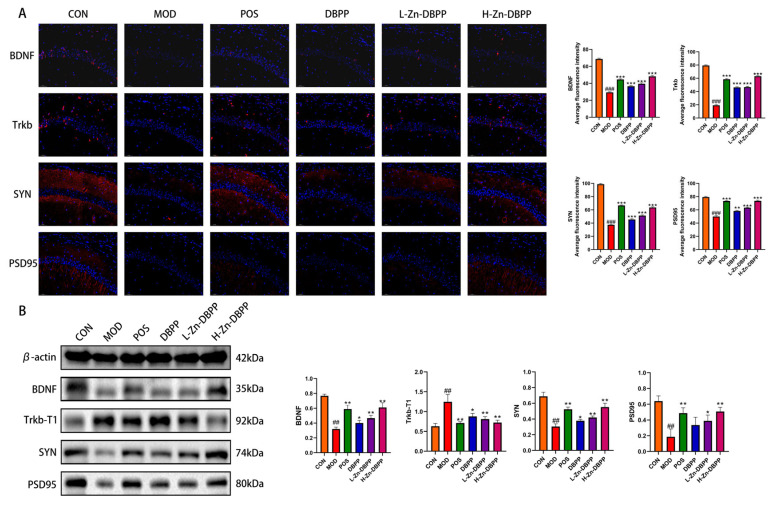
(**A**) Immunofluorescence images of the mouse hippocampal CA1 region and bar graphs depicting the fluorescence intensity of BDNF, TrkB, SYN, and PSD95 proteins (*n* = 3). (**B**) Western blot bands of BDNF, TrkB, SYN, and PSD95, along with bar graphs showing the densitometric gray values of each protein (*n* = 3). (## *p* < 0.01 ### *p* < 0.001 vs. CON group. * *p* < 0.05, ** *p* < 0.01, *** *p* < 0.001 vs. MOD group).

Figure 5A shows the immunofluorescence pharmacodynamic results. In the BDNF immunofluorescence assay, the fluorescence intensity in the MOD group was significantly decreased compared with the CON group (*p* < 0.001). Relative to the MOD group, both DBPP and Zn-DBPP treatments increased the fluorescence intensity, with the H-Zn-DBPP group exhibiting the most pronounced effect (*p* < 0.001). Similarly, for TrkB immunofluorescence, the MOD group showed a significant reduction in fluorescence intensity versus the CON group (*p* < 0.001), and both DBPP and Zn-DBPP treatments increased intensity compared with the MOD group, with the H-Zn-DBPP group again demonstrating the most significant effect (*p* < 0.001). In the SYN immunofluorescence assay, the fluorescence intensity in the MOD group was significantly lower than that in the CON group (*p* < 0.001); both DBPP and Zn-DBPP treatments elevated the intensity relative to the MOD group, and the H-Zn-DBPP group showed the most remarkable increase (*p* < 0.001). For PSD95 immunofluorescence, the MOD group exhibited a significant decline in fluorescence intensity compared with the CON group (*p* < 0.001), whereas DBPP and Zn-DBPP treatments increased the intensity, with the H-Zn-DBPP group again achieving the most significant effect (*p* < 0.001).

Figure 5B presents the Western blot results. For BDNF, the gray value in the MOD group was significantly lower than that in the CON group (*p* < 0.01). Compared with the MOD group, both DBPP and Zn-DBPP treatments significantly increased the gray values, and the H-Zn-DBPP group showed the most prominent effect (*p* < 0.01). For TrkB-T1, the gray value in the MOD group was significantly elevated relative to the CON group (*p* < 0.01); DBPP and Zn-DBPP treatments significantly reduced the gray values compared with the MOD group, with the H-Zn-DBPP group being the most effective (*p* < 0.01). For SYN, the gray value in the MOD group was significantly decreased versus the CON group (*p* < 0.01); both DBPP and Zn-DBPP treatments markedly increased the gray values, and the H-Zn-DBPP group exhibited the most significant elevation (*p* < 0.01). For PSD95, the gray value in the MOD group was significantly lower than that in the CON group (*p* < 0.01); DBPP and Zn-DBPP treatments significantly increased the gray values relative to the MOD group, with the H-Zn-DBPP group again showing the most significant effect (*p* < 0.01).

Immunofluorescence and Western blot results collectively indicate that DBPP and Zn-DBPP may ameliorate memory impairment induced by insomnia in mice, through a mechanism involving the upregulation of BDNF, TrkB, SYN, and PSD95 protein expression levels, along with the downregulation of TrkB-T1 protein expression.

#### 3.9.2. Result of Mechanism Investigation Experiment

**Figure 6:**
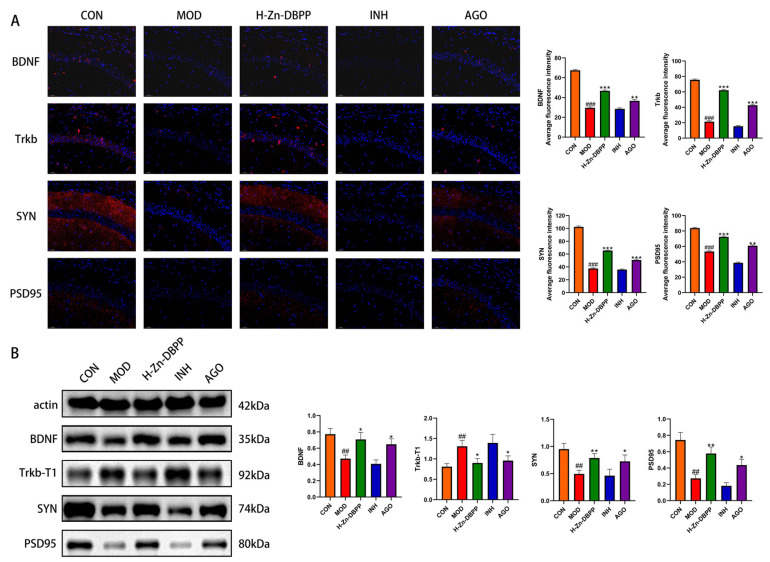
(**A**) In mechanistic experiments, immunofluorescence images of the mouse hippocampal CA1 region and bar graphs showing the fluorescence intensities of BDNF, TrkB, SYN, and PSD95 proteins (*n* = 3). (**B**) Western blot bands of BDNF, TrkB, SYN, and PSD95 from mechanistic experiments, along with bar graphs showing the densitometric gray values for each protein (*n* = 3). (## *p* < 0.01 ### *p* < 0.001 vs. CON group. * *p* < 0.05, ** *p* < 0.01, *** *p* < 0.001 vs. MOD group).

Figure 6A presents the immunofluorescence results. For BDNF, compared with the CON group, the fluorescence intensity in the MOD group was significantly decreased (*p* < 0.001). Compared with the MOD group, the fluorescence intensity was significantly increased in both the AGO group (*p* < 0.01) and the H-Zn-DBPP group (*p* < 0.001), indicating that Zn-DBPP promotes BDNF protein expression. For TrkB, compared with the CON group, the fluorescence intensity in the MOD group was significantly decreased (*p* < 0.001). Compared with the MOD group, the fluorescence intensity was significantly increased in both the AGO group (*p* < 0.001) and the H-Zn-DBPP group (*p* < 0.001), indicating that Zn-DBPP promotes TrkB expression. For SYN, compared with the CON group, the fluorescence intensity in the MOD group was significantly decreased (*p* < 0.001). Compared with the MOD group, the fluorescence intensity was significantly increased in both the AGO group (*p* < 0.001) and the H-Zn-DBPP group (*p* < 0.001), confirming that Zn-DBPP promotes SYN expression. For PSD95, compared with the CON group, the fluorescence intensity in the MOD group was significantly decreased (*p* < 0.001). Compared with the MOD group, the fluorescence intensity was significantly increased in both the AGO group (*p* < 0.01) and the H-Zn-DBPP group (*p* < 0.001), indicating that Zn-DBPP promotes PSD95 expression.

Figure 6B presents the Western blot results. For BDNF, the gray value in the MOD group was significantly decreased compared with the CON group (*p* < 0.01), consistent with the INH group, indicating that PCPA may inhibit BDNF expression. Compared with the MOD group, the gray values in both the AGO group (*p* < 0.05) and the H-Zn-DBPP group (*p* < 0.05) were significantly increased, suggesting that Zn-DBPP may promote BDNF expression. For TrkB-T1, the gray value in the MOD group was significantly increased compared with the CON group (*p* < 0.01), consistent with the INH group, indicating that PCPA may promote TrkB-T1 expression. Compared with the MOD group, the gray values in both the AGO group (*p* < 0.05) and the H-Zn-DBPP group (*p* < 0.05) were significantly decreased, suggesting that Zn-DBPP may inhibit TrkB-T1 expression. For SYN, the gray value in the MOD group was significantly decreased compared with the CON group (*p* < 0.01), consistent with the INH group, indicating that PCPA may inhibit SYN expression. Compared with the MOD group, the gray values in both the AGO group (*p* < 0.05) and the H-Zn-DBPP group (*p* < 0.01) were significantly increased, suggesting that Zn-DBPP may promote SYN expression. For PSD95, the gray value in the MOD group was significantly decreased compared with the CON group (*p* < 0.01), consistent with the INH group, indicating that PCPA may inhibit PSD95 expression. Compared with the MOD group, the gray values in both the AGO group (*p* < 0.05) and the H-Zn-DBPP group (*p* < 0.01) were significantly increased, suggesting that Zn-DBPP may promote PSD95 expression.

Mechanistic experimental results from immunofluorescence and Western blotting further suggest that Zn-DBPP may exert a regulatory effect on the BDNF/TrkB pathway. Its mechanism of action may involve upregulating this pathway to enhance the expression of BDNF, TrkB, SYN, and PSD95 proteins, while simultaneously suppressing the expression of TrkB-T1. These proteins are closely associated with memory function. Zn-DBPP improved memory impairment in PCPA-induced insomniac mice by affecting the expression of this pathway and its related proteins.

## 4. Discussion

In this experiment, the enzymatic hydrolysis method for DBPP and the chelation method for Zn-DBPP were optimized. Characterization analysis indicated that zinc was successfully coordinated with the peptide to form a chelate. Experimental results showed that both DBPP and Zn-DBPP exhibited excellent antioxidant capacity, with Zn-DBPP demonstrating superior antioxidant activity compared to DBPP, and this finding is consistent with previous studies [35]. In this experiment, the hydrolysis degree of the peptides was determined by the ph-stat method. This method is frequently used in the determination of the hydrolysis degree of peptides prepared by enzymatic hydrolysis. It has the advantages of high accuracy and a simple calculation method [36]. During the chelation process in the present study, the EDTA method was employed to determine the zinc content in Zn-DBPP, which is a widely adopted approach for the determination of zinc in chelates. [37]. In the optimization process of enzymatic hydrolysis and chelation methods, this study employed a combination of single-factor experiments and response surface methodology (RSM) to optimize the enzymatic hydrolysis and chelation processes. Response surface experiments enable more precise optimization of the optimal process conditions and validate the single-factor experimental results. This approach has been widely used in previous studies and offers the advantage of high data accuracy [38]. In future experiments, we will simulate industrial production environments and conditions to further optimize the processes of enzymatic hydrolysis and chelation, thereby laying a foundation for subsequent product development and industrial production.

In this experiment, an animal model of insomnia was established using the PCPA method. This approach relies on the irreversible inhibition of tryptophan hydroxylase (TPH) by PCPA, which specifically blocks the central synthesis of 5-hydroxytryptamine (5-HT). The consequent rapid depletion of brain 5-HT levels disrupts the homeostasis of the sleep–wake cycle, thereby producing a physiological state that closely resembles clinical primary insomnia [39]. Compared with non-specific models induced by physical stimulation or drugs, this PCPA-based method precisely targets the key sleep-regulatory target 5-HT, while avoiding interference with other neurotransmitter systems such as dopamine and norepinephrine. This specificity thereby provides a clearer understanding of the pathogenesis of insomnia [40]. Furthermore, PCPA inhibits 5-HT biosynthesis by selectively blocking tryptophan hydroxylase. This mechanism induces multi-level neuropathological changes in hippocampal structure and function, ultimately leading to significant impairment of learning and memory abilities [41]. Moreover, depletion of central 5-HT further reduces brain-derived neurotrophic factor (BDNF) levels in the prefrontal cortex and hippocampus, thereby weakening synaptic plasticity and increasing the susceptibility of hippocampal neurons to damage [42]. In conclusion, the PCPA-induced insomnia model impairs BDNF-mediated synaptic plasticity through 5-HT depletion, thereby disrupting the integrity of neural circuits essential for memory formation and consolidation. This establishes a causal relationship between insomnia and memory disorders.

This study is the first to demonstrate that DBPP and Zn-DBPP significantly improve insomnia-induced memory impairment in mice. The underlying mechanism may involve regulation of the BDNF/TrkB signaling pathway. Specifically, both compounds effectively upregulate the expression of brain-derived neurotrophic factor (BDNF) and its high-affinity receptor TrkB in the hippocampus, thereby enhancing the expression of downstream synaptic proteins, including synaptophysin (SYN) and postsynaptic density protein 95 (PSD95). These improvements may be attributed to the abundant glutamate content in DBPP and Zn-DBPP. Glutamate is relatively abundant in the mammalian brain and plays a pivotal role in the central nervous system by regulating synaptic activity, maintaining neural network stability, and serving as a key mediator of neuronal damage and inflammatory responses [43]. In this experiment, the glutamate content in DBPP was 15.43%, while that in Zn-DBPP increased to 16.94% following chelation. This combination of high glutamate levels and high zinc ion concentration may exert more effective regulatory effects on the nervous system and help maintain its homeostasis [44]. This may explain why Zn-DBPP exhibited better therapeutic efficacy than DBPP.

In the present study, both DBPP and Zn-DBPP exhibited favorable therapeutic effects; however, certain limitations remain that warrant further investigation in future advanced experiments. To address these issues, we plan to include a ZnSO_4_ treatment group in our subsequent studies. Although clinically used zinc supplements for memory impairment—such as zinc aspartate and zinc gluconate—do not include zinc sulfate, the addition of a ZnSO_4_ control group is methodologically important to rule out any potential confounding effects of residual ZnSO_4_ on the efficacy evaluation [45]. Moreover, excessive intake of zinc sulfate may impair memory and disrupt sleep, rendering it unsuitable for ameliorating memory deficits caused by insomnia [46,47,48]. However, some studies have reported that zinc sulfate exerts certain beneficial effects on some neurological disorders [49]. Nevertheless, the potential influence suggested by the aforementioned literature cannot be overlooked. Therefore, in future experiments, we plan to further investigate whether trace amounts of ZnSO_4_ may interfere with the therapeutic efficacy of Zn-DBPP. In parallel, we will conduct gastrointestinal simulation experiments to compare the stability of ZnSO_4_ and Zn-DBPP in the digestive tract, thereby providing a theoretical basis for future clinical translation. Our current results indicate that Zn-DBPP exhibits stronger antioxidant activity than DBPP; however, whether this enhanced activity is attributable to zinc coordination per se or to conformational/ compositional changes in the peptide following chelation remains to be elucidated in subsequent studies. Regarding brain distribution, although we observed an increase in cerebral zinc content following Zn-DBPP administration, this finding alone does not constitute direct evidence of brain penetration. In future work, we will employ mass spectrometry imaging (MSI) to map the distribution of peptides in mouse brain tissue and systematically evaluate whether Zn-DBPP can cross the blood–brain barrier, so as to more accurately determine its actual accessibility to the central nervous system. Furthermore, the characterization data obtained in the present study merely confirm successful chelation between zinc ions and the peptide, but do not reveal the underlying chelation mechanism, coordination mode, or coordination environment. To address these gaps, we will utilize circular dichroism (CD) spectroscopy, nuclear magnetic resonance (NMR), and electrospray ionization mass spectrometry (ESI-MS) for further structural identification of the chelate, and will determine the peptide chain length and conformation of DBPP, as well as the peptide-to-zinc stoichiometric ratio within Zn-DBPP. In addition, we plan to conduct a comprehensive investigation into the coordination environment and its influence on the biological activity of Zn-DBPP.

In recent years, food-derived peptide–zinc chelates have attracted considerable attention due to their potential in neuroprotection. Zhang et al. (2023) demonstrated that a pine nut peptide–zinc chelate could improve learning and memory abilities in mice by modulating the activities of acetylcholinesterase and choline acetyltransferase [50]. Yu et al. (2025) reported that the oyster peptide-chitooligosaccharide-zinc complex (OCZn) restored hippocampal neuronal integrity and upregulated the expression of PSD95 and α-Syn in a zinc-deficient mouse model [51]. Zhao et al. (2024) further confirmed that the walnut peptide–zinc chelate exhibited superior antioxidant and glycemic regulation activities compared with the unchelated peptide [52]. Collectively, the above studies indicate that zinc chelation can enhance the bioactivity of peptides, which is consistent with our finding that Zn-DBPP exhibited stronger DPPH and ABTS radical-scavenging activities than DBPP. With regard to deer-derived products, deer antler peptides have been shown to ameliorate D-galactose-induced brain injury through upregulation of brain-derived neurotrophic factor (BDNF) and vascular endothelial growth factor (VEGF) expression [53]. However, studies on zinc chelation using deer brain as a peptide source have not been reported to date. In this study, we prepared a peptide–zinc chelate from deer brain for the first time and demonstrated that it ameliorated memory impairment in insomnia-induced mice, possibly through upregulation of the BDNF-TrkB signaling pathway. This finding not only enriches the functional research on deer-derived products but also provides a novel candidate for the treatment of cognitive impairment caused by insomnia.In future research, we plan to further develop DBPP and Zn-DBPP into functional food products, such as candies and beverages, in order to improve their flavor and enhance consumer acceptability.Historically, deer-based products were accessible only to royalty due to the scarcity of deer resources. However, with the rapid development of modern animal husbandry, deer production has increased substantially, ensuring a more abundant supply of raw materials for product development. Moreover, there is currently no significant cultural resistance to deer-related products, which represents an advantage for their commercialization. We aim to capitalize on this advantage to gain greater opportunities in future product development and production.

In this experiment, both DBPP and Zn-DBPP effectively ameliorated insomnia-induced memory impairment, with Zn-DBPP demonstrating superior efficacy compared to DBPP and achieving effects nearly identical to those of Zhennaoning. However, the present study did not examine the preventive potential of these peptides against insomnia. Given the escalating pressures and accelerating pace of modern life, it is increasingly important not only to treat existing insomnia but also to proactively prevent the onset of sleep-related symptoms. In future research, we plan to further investigate the prophylactic effects of deer brain peptides and their zinc chelate on insomnia, as well as their viability as functional health supplements for alleviating work-related fatigue and enhancing daily cognitive performance. This work not only provides a theoretical basis for subsequent clinical research and industrial production, but also contributes to the development of deer-derived peptides.

## 5. Conclusions

In conclusion, this study successfully established an efficient enzymatic hydrolysis process for preparing deer brain peptides and the zinc chelation conditions. The obtained Zn-DBPP exhibits excellent antioxidant activity and neuroprotective effects. In vivo experiments confirmed that Zn-DBPP can improve memory impairment in PCPA-induced sleep-deprived mice by regulating the BDNF/TrkB pathway and alleviating hippocampal neuron damage. This study provides a scientific basis for the deep processing and utilization of deer brain resources and lays an experimental foundation for developing functional foods or drug candidates that improve sleep-related cognitive dysfunction.

## Figures and Tables

**Table 1:** Experimental design results of single-enzyme response surface.

Test Number	A: Temperature (°C)	B: pH	C: Enzyme Dosage (U/g)	D: Time (h)	DH (%)
1	−1	−1	0	0	38.26
2	1	−1	0	0	38.42
3	−1	1	0	0	37.43
4	1	1	0	0	38.54
5	0	0	−1	−1	35.92
6	0	0	1	−1	40.77
7	0	0	−1	1	39.68
8	0	0	1	1	43.64
9	−1	0	0	−1	37.25
10	1	0	0	−1	39.08
11	−1	0	0	1	41.95
12	1	0	0	1	41.68
13	0	−1	−1	0	36.34
14	0	1	−1	0	35.73
15	0	−1	1	0	38.85
16	0	1	1	0	38.79
17	−1	0	−1	0	38.05
18	1	0	−1	0	39.26
19	−1	0	1	0	42.48
20	1	0	1	0	42.06
21	0	−1	0	−1	35.64
22	0	1	0	−1	35.48
23	0	−1	0	1	40.23
24	0	1	0	1	37.74
25	0	0	0	0	46.33
26	0	0	0	0	45.98
27	0	0	0	0	46.27

**Table 2:** Regression model and analysis of variance.

Source	Sum of Squares	*df*	Equal Square	F-Value	*p*-Value	Significance
Model	263.61	14	18.83	83.07	<0.0001	Significant
A	1.09	1	1.09	4.82	0.0486	
B	1.35	1	1.35	5.97	0.031	
C	38.92	1	38.92	171.69	<0.0001	
D	35.98	1	35.98	158.76	<0.0001	
AB	0.2256	1	0.2256	0.9954	0.3381	
AC	0.6642	1	0.6642	2.93	0.1126	
AD	1.1	1	1.1	4.86	0.0477	
BC	0.0756	1	0.0756	0.3337	0.5742	
BD	1.36	1	1.36	5.99	0.0308	
CD	0.198	1	0.198	0.8737	0.3684	
A2	38.18	1	38.18	168.43	<0.0001	
B2	164.38	1	164.38	725.22	<0.0001	
C2	49.18	1	49.18	216.98	<0.0001	
D2	59.87	1	59.87	264.13	<0.0001	
Residual	2.72	12	0.2267			
Lack of Fit	2.65	10	0.265	7.56	0.1223	Not significant
Pure Error	0.0701	2	0.035			
Cor Total	266.33	26				

Note: *p* < 0.01 is extremely significant, *p*-value < 0.05 is significant, *df* is degrees of freedom.

**Table 3:** Experimental design results of chelated zinc response surface.

Test Number	A: pH	B: Temperature (°C)	C: Time (min)	DH (%)
1	6	60	10	93.26
2	8	60	10	114.15
3	6	80	10	108.37
4	8	80	10	121.35
5	6	70	5	106.58
6	8	70	5	125.86
7	6	70	20	112.17
8	8	70	20	131.89
9	7	60	5	102.36
10	7	80	5	112.53
11	7	60	20	104.43
12	7	80	20	126.91
13	7	70	10	140.45
14	7	70	10	136.94
15	7	70	10	138.23

**Table 4:** Regression model and analysis of variance of chelation experiment.

Source	Sum of Squares	*df*	Mean Square	F-Value	*p*-Value	Significance
Model	2941.24	9	326.8	97.45	<0.0001	significant
A-pH	637.66	1	637.66	190.15	<0.0001	
B-temperature	418.87	1	418.87	124.91	0.0001	
C-time	98.49	1	98.49	29.37	0.0029	
AB	15.64	1	15.64	4.66	0.0832	
AC	0.3972	1	0.3972	0.1184	0.7447	
BC	46.64	1	46.64	13.91	0.0136	
A2	434.27	1	434.27	129.5	<0.0001	
B2	1251.77	1	1251.77	373.28	<0.0001	
C2	333.17	1	333.17	99.35	0.0002	
Residual	16.77	5	3.35			
Lack of Fit	10.46	3	3.49	1.11	0.5071	not significant
Pure Error	6.3	2	3.15			
Cor Total	2958.01	14				

Note: *p* < 0.01 is extremely significant, *p*-value < 0.05 is significant, *df* is degrees of freedom.

**Table 5:** The amino acid composition of DBPP and Zn-DBPP.

Types of Amino Acids	The Content of Each Amino Acid in DBPP	The Content of Each Amino Acid in Zn-DBPP
Asp	10.02%	11.69%
Thr	5.22%	4.61%
Ser	5.73%	6.42%
Glu	15.43%	16.94%
Gly	5.53%	5.77%
Ala	6.24%	5.01%
Cys	1.69%	4.60%
Val	5.03%	4.37%
Met	2.08%	1.79%
Ile	4.00%	2.98%
Leu	8.10%	5.64%
Tyr	4.58%	2.82%
Phe	5.77%	3.72%
His	3.09%	4.01%
Lys	7.35%	8.48%
Arg	5.70%	7.04%
Pro	4.45%	4.12%
Total content	679.34 (mg/g)	532.57 (mg/g)

## Data Availability

The datasets presented in this article are not readily available because the data are part of an ongoing study.

## Abbreviations

The following abbreviations are used in this manuscript:

DBPP	deer brain peptides
Zn-DBPP	deer brain peptide chelated with zinc
PCPA	para-chlorophenylalanine
KM	Kunming (mice)
FTIR	Fourier transform infrared spectroscopy
UV-Vis	ultraviolet–visible spectroscopy
SEM	scanning electron microscopy
AAS	atomic absorption spectrometry
ELISA	enzyme-linked immunosorbent assay
HE	hematoxylin and eosin
BDNF	brain-derived neurotrophic factor
TrkB	tropomyosin receptor kinase B
SYN	synaptophysin
PSD95	postsynaptic density protein 95
DPPH	2,2-diphenyl-1-picrylhydrazyl
TCM	Traditional Chinese Medicine
ABTS	2,2′-azino-bis(3-ethylbenzothiazoline-6-sulfonic acid)
RSM	response surface methodology
ZnSO_4_	zinc sulfate
RIPA	radio-immunoprecipitation assay lysis buffer
PMSF	phenylmethylsulfonyl fluoride
EDTA	ethylenediaminetetraacetic acid
BSA	bovine serum albumin
PBS	phosphate-buffered saline
SPF	specific pathogen-free
WB	Western blot
TPH	tryptophan hydroxylase
5-HT	5-hydroxytryptamine (serotonin)
HRP	horseradish peroxidase
ECL	enhanced chemiluminescence
ANOVA	analysis of variance
DH	degree of hydrolysis
CON	control group
MOD	model group
POS	positive drug group
L-Zn-DBPP	low-dose zinc–deer brain peptide group
H-Zn-DBPP	high-dose zinc–deer brain peptide group
INH	inhibitor group (TrkB receptor antagonist Ana-12)
AGO	agonist group (7,8-dihydroxyflavone)

## References

[1] Benjafield A.V., Sert Kuniyoshi F.H., Malhotra A., Martin J.L., Morin C.M., Maurer L.F., Cistulli P.A., Pépin J.-L., Wickwire E.M. (2025). Estimation of the global prevalence and burden of insomnia: A systematic literature review-based analysis. Sleep Med. Rev..

[2] Zhai L., Shen H., Wu S., Guo L., Yang Y., Sheng J., Han C. (2025). Deer antler peptidess inhibit microglial activation via TREM2 to improve behavior and neuroinflammation in CUMS mice. Int. Immunopharmacol..

[3] Olaithe M., Ree M., McArdle N., Donaldson S., Pushpanathan M., Eastwood P.R., Bucks R.S. (2021). Cognitive Dysfunction in Insomnia Phenotypes: Further Evidence for Different Disorders. Front. Psychiatry.

[4] Zhao J.L., Cross N., Yao C.W., Carrier J., Postuma R.B., Gosselin N., Kakinami L., Dang-Vu T.T. (2022). Insomnia disorder increases the risk of subjective memory decline in middle-aged and older adults: A longitudinal analysis of the Canadian Longitudinal Study on Aging. Sleep.

[5] Raven F., Van der Zee E.A., Meerlo P., Havekes R. (2018). The role of sleep in regulating structural plasticity and synaptic strength: Implications for memory and cognitive function. Sleep Med. Rev..

[6] Sarma A., Havekes R., Wixted J. (2025). Sleep and memory: Elucidating the effects of sleep deprivation on different types of memory. Learning and Memory: A Comprehensive Reference.

[7] Zhang L., Guo Z., Han Y., Yan H., Lv D., Yao P., Zhao J., Chen L., Guo W. (2025). Altered cerebral functional activity and its associated genetic profiles underlying chronic insomnia disorder before and after treatment. World J. Biol. Psychiatry.

[8] Lu C., Geng Y., Guan X., Meng Y., Zhu M., Zhao Y. (2025). Adverse events of pharmacological interventions for insomnia disorder in adults: A systematic review and network meta-analysis. Front. Psychiatry.

[9] Aiello G., Cropotova J., Kvangarsnes K., d’Adduzio L., Fanzaga M., Bollati C., Boschin G., Roda G., Lammi C. (2025). Ultrasonicated Atlantic herring side streams as source of multifunctional bioactive and bioavailable peptides. npj Sci. Food.

[10] Yan X., Wu Q., Pei Q., Zhang S., Ji C., Chen Y., Dai Y., Zhu B., Lin X. (2025). Binding mechanism, digestive stability, and cellular interactions of a potential organic Iron supplement: Fermented scallop skirt peptide (DDDHPGIF)-Iron complex. Food Res. Int..

[11] Zhao Q., Liang W., Xiong Z., Li C., Zhang L., Rong J., Xiong S., Liu R., You J., Yin T. (2024). Digestion and absorption characteristics of iron-chelating silver carp scale collagen peptide and insights into their chelation mechanism. Food Res. Int..

[12] López-Guerrero V.E., Posadas Y., Sánchez-López C., Smart A., Miranda J., Singewald K., Bandala Y., Juaristi E., Den Auwer C., Perez-Cruz C. (2025). A Copper-Binding Peptide with Therapeutic Potential against Alzheimer’s Disease: From the Blood-Brain Barrier to Metal Competition. ACS Chem. Neurosci..

[13] Guo M., Huang L., Li Y., Kong W., Liu Y., Jiang X., Sun J. (2025). Characterization and binding site profiling of zinc-chelated peptides from Takifugu rubripes bone with enhanced biological activity. Food Chem..

[14] Samimiazad A., Mirdamadi S., Akhavan Sepahi A., Sedaghati M., Safavi M. (2025). Characterization of zinc-chelating peptides prepared from Arthrospira platensis proteins. Food Biosci..

[15] Umesh S.B., Sadanandan B., Marabanahalli Yogendraiah K., Vijayalakshmi V. (2026). The Impact of Zinc on Cellular Dynamics, Brain Function, and its Therapeutic Potential in Neuronal Regeneration. Mol. Neurobiol..

[16] Lu D., Peng M., Yu M., Jiang B., Wu H., Chen J. (2021). Effect of Enzymatic Hydrolysis on the Zinc Binding Capacity and in vitro Gastrointestinal Stability of Peptides Derived From Pumpkin (*Cucurbita pepo* L.) Seeds. Front. Nutr..

[17] Tsuji K. (1973). Application of a pH-stat to ultramicrohydrogenation analysis. Microchem. J..

[18] Tibbetts S.M., Milley J.E., Ross N.W., Verreth J.A.J., Lall S.P. (2011). In vitro pH-Stat protein hydrolysis of feed ingredients for Atlantic cod, Gadus morhua. 1. Development of the method. Aquaculture.

[19] Yang S., Liu T., Chen W., Zong Y., Geng J., Zhao Y., He Z., Du R. (2025). Preparation, Characterization, and In Vitro Stability Analysis of Deer Sinew Peptide-Zinc Chelate. Foods.

[20] van Slageren R., den Boef G., van der Linden W.E. (1973). Determination of traces of zinc by fluorimetrically indicated complexometric titrations. Talanta.

[21] Baliyan S., Mukherjee R., Priyadarshini A., Vibhuti A., Gupta A., Pandey R.P., Chang C.M. (2022). Determination of Antioxidants by DPPH Radical Scavenging Activity and Quantitative Phytochemical Analysis of Ficus religiosa. Molecules.

[22] Terfi S., Djerrad Z., Krimat S., Sadi F. (2023). Phytochemical composition, cytotoxicity, antioxidant and antimicrobial responses of Lavandula dentata L. grown under different levels of heavy metals stress condition. Drug Chem. Toxicol..

[23] Yang J., Shi J., Zhou Y., Zou Y., Xu W., Xia X., Wang D. (2024). Preparation, Characterization and Stability of Calcium-Binding Peptides Derived from Chicken Blood. Foods.

[24] Luo J., Yao X., Soladoye O.P., Zhang Y., Fu Y. (2022). Phosphorylation modification of collagen peptides from fish bone enhances their calcium-chelating and antioxidant activity. LWT.

[25] Huang H., Fu M., Chen M. (2019). Preparation, Characteristics, and Formation Mechanism of Oyster Peptide-Zinc Nanoparticles. J. Ocean Univ. China.

[26] Zhang F., Zhang X., Peng Q., Tang L. (2023). Electroacupuncture of the cymba concha alleviates p-chlorophenylalanine-induced insomnia in mice. Acupunct. Med..

[27] Fang S., Xi H., Zhang K., Fang X., Yang Y., Li J., Yang W. (2026). Zhinao Capsule improves learning and memory impairment in APP/PS1 mice through gut-brain axis-mediated inhibition of neuroinflammation. Front. Microbiol..

[28] Shen C.-Y., Li X.-Y., Ma P.-Y., Li H.-L., Xiao B., Cai W.-F., Xing X.-F. (2022). Nicotinamide mononucleotide (NMN) and NMN-rich product supplementation alleviate p-chlorophenylalanine-induced sleep disorders. J. Funct. Foods.

[29] Vorhees C.V., Williams M.T. (2006). Morris water maze: Procedures for assessing spatial and related forms of learning and memory. Nat. Protoc..

[30] Cardiff R.D., Miller C.H., Munn R.J. (2014). Manual hematoxylin and eosin staining of mouse tissue sections. Cold Spring Harb. Protoc..

[31] Plata A., Lebedeva A., Denisov P., Nosova O., Postnikova T.Y., Pimashkin A., Brazhe A., Zaitsev A.V., Rusakov D.A., Semyanov A. (2018). Astrocytic Atrophy Following Status Epilepticus Parallels Reduced Ca(^2+^) Activity and Impaired Synaptic Plasticity in the Rat Hippocampus. Front. Mol. Neurosci..

[32] Lupaescu A.V., Jureschi M., Ciobanu C.I., Ion L., Zbancioc G., Petre B.A., Drochioiu G. (2019). FTIR and MS Evidence for Heavy Metal Binding to Anti-amyloidal NAP-Like Peptides. Int. J. Pept. Res. Ther..

[33] Zhao J., Dong T., Yu P., Wang J. (2022). Conformation and Metal Cation Binding of Zwitterionic Alanine Tripeptide in Saline Solutions by Infrared Vibrational Spectroscopy and Molecular Dynamics Simulations. J. Phys. Chem. B.

[34] Zhang L., Wang Y., Ni Y., Zhang Y., Liu Y., Du B., Luo H., Lin D. (2024). Production of novel mung bean peptides-based zinc supplement: Process optimization, chelation mechanism, and stability assessment in vitro. Food Biosci..

[35] Zhao F., Hou W., Guo L., Wang C., Liu Y., Liu X., Min W. (2024). Novel strategy to the characterization and enhance the glycemic control properties of walnut-derived peptides via zinc chelation. Food Chem..

[36] Beaubier S., Framboisier X., Ioannou I., Galet O., Kapel R. (2019). Simultaneous quantification of the degree of hydrolysis, protein conversion rate and mean molar weight of peptides released in the course of enzymatic proteolysis. J. Chromatogr. B.

[37] Suzuki T., Tiwari D., Hioki A. (2007). Precise Chelatometric Titrations of Zinc, Cadmium, and Lead with Molecular Spectroscopy. Anal. Sci..

[38] Zhang M., Mu T.-H. (2016). Optimisation of antioxidant hydrolysate production from sweet potato protein and effect of in vitro gastrointestinal digestion. Int. J. Food Sci. Technol..

[39] Wang L., Qi X., Wang S., Tian C., Zou T., Liu Z., Chen Q., Chen Y., Zhao Y., Li S. (2024). Banxia-Yiyiren alleviates insomnia and anxiety by regulating the gut microbiota and metabolites of PCPA-induced insomnia model rats. Front. Microbiol..

[40] Li Q., Zhai J., Du T., He J., Zhang J., Tian Y., Ma K., Si J., Yin J., Li Y. (2025). Ferulic acid methylester improves the comorbidity of insomnia and anxiety in a rat model of PCPA-induced sleep disorder by activating DRN 5-HT neurons. Neurosci. Lett..

[41] Mashahadi Z., Saadati H., Ghaheri Fard S. (2024). Early-life manipulation of the serotonergic system exacerbates the harmful effects of sleep deprivation on cognitive functions. Int. J. Dev. Neurosci..

[42] van Donkelaar E.L., van den Hove D.L.A., Blokland A., Steinbusch H.W.M., Prickaerts J. (2009). Stress-mediated decreases in brain-derived neurotrophic factor as potential confounding factor for acute tryptophan depletion-induced neurochemical effects. Eur. Neuropsychopharmacol..

[43] Nicosia N., Giovenzana M., Misztak P., Mingardi J., Musazzi L. (2024). Glutamate-Mediated Excitotoxicity in the Pathogenesis and Treatment of Neurodevelopmental and Adult Mental Disorders. Int. J. Mol. Sci..

[44] Paoletti P., Vergnano A.M., Barbour B., Casado M. (2009). Zinc at glutamatergic synapses. Neuroscience.

[45] Piacenza F., Giacconi R., Costarelli L., Malavolta M. (2023). Preliminary Comparison of Fractional Absorption of Zinc Sulphate, Zinc Gluconate, and Zinc Aspartate after Oral Supple-Mentation in Healthy Human Volunteers. Nutrients.

[46] Yang Y., Jing X.P., Zhang S.P., Gu R.X., Tang F.X., Wang X.L., Xiong Y., Qiu M., Sun X.Y., Ke D. (2013). High dose zinc supplementation induces hippocampal zinc deficiency and memory impairment with inhibition of BDNF signaling. PLoS ONE.

[47] Kong X., Liu L., Sheng X. (1998). Effects of excessive zinc in fodder on brain development and abilities of learning and memory and their mechanisms in young rats. Zhonghua Yu Fang. Yi Xue Za Zhi.

[48] Takeuchi H., Taki Y., Nouchi R., Yokoyama R., Kotozaki Y., Nakagawa S., Sekiguchi A., Iizuka K., Hanawa S., Araki T. (2020). Succeeding in deactivating: Associations of hair zinc levels with functional and structural neural mechanisms. Sci. Rep..

[49] Zhang T., Chen Q. (2026). Metabolomics study of the effects of zinc sulfate in minimal hepatic encephalopathy. Sci. Rep..

[50] Zhang Z., Sun J., Li Y., Yang K., Wei G., Zhang S. (2023). Ameliorative effects of pine nut peptide-zinc chelate (*Korean pine*) on a mouse model of Alzheimer’s disease. Exp. Gerontol..

[51] Yu X., Liu R., Xu Y., Pan N., Liu X., Nakamura Y., Zhou D. (2025). Mechanism of TGase-induced oyster peptide-chitosan oligosaccharide glycosylation and amelioration effects of its zinc complex on zinc deficiency-associated neuron damages and oxidative stress. Food Res. Int..

[52] Zhao S., Wang L., Liang J., Jin F., Wang F. (2024). Preparation, characterization and microencapsulation of walnut (*Juglans regia* L.) peptides-zinc chelate. J. Food Sci..

[53] Chen S., Zong Y., Li J., He Z., Du R. (2025). Protective Effects of Deer Antler Peptides on D-Galactose-Induced Brain Injury. Nutrients.

